# *LocText*: relation extraction of protein localizations to assist database curation

**DOI:** 10.1186/s12859-018-2021-9

**Published:** 2018-01-17

**Authors:** Juan Miguel Cejuela, Shrikant Vinchurkar, Tatyana Goldberg, Madhukar Sollepura Prabhu Shankar, Ashish Baghudana, Aleksandar Bojchevski, Carsten Uhlig, André Ofner, Pandu Raharja-Liu, Lars Juhl Jensen, Burkhard Rost

**Affiliations:** 1 Bioinformatics & Computational Biology, Department of Informatics, Technical University of Munich (TUM), Boltzmannstr. 3, Garching, 85748 Germany; 2 Microsoft, Microsoft Development Center Copenhagen, Kanalvej 7, Kongens Lyngby, 2800 Denmark; 3 Department of Computer Science and Information Systems, Birla Institute of Technology and Science K. K. Birla Goa Campus, Zuarinagar, 403726 Goa India; 4 Novo Nordisk Foundation Center for Protein Research, Faculty of Health and Medical Sciences, University of Copenhagen, Copenhagen N, 2200 Denmark; 5 Institute for Advanced Study (TUM-IAS), Lichtenbergstr. 2a, Garching/Munich, 85748 Germany; 6 TUM School of Life Sciences Weihenstephan (WZW), Alte Akademie 8, Freising, Germany; 7 Columbia University, Department of Biochemistry and Molecular Biophysics, Columbia University, New York, USA; 8 New York Consortium on Membrane Protein Structure (NYCOMPS), 701 West, 168th Street, New York, 10032 NY USA

**Keywords:** Relation extraction, Text mining, Protein, Subcellular localization, GO, Annotations, Database curation

## Abstract

**Background:**

The subcellular localization of a protein is an important aspect of its function. However, the experimental annotation of locations is not even complete for well-studied model organisms. Text mining might aid database curators to add experimental annotations from the scientific literature. Existing extraction methods have difficulties to distinguish relationships between proteins and cellular locations co-mentioned in the same sentence.

**Results:**

*LocText* was created as a new method to extract protein locations from abstracts and full texts. *LocText* learned patterns from syntax parse trees and was trained and evaluated on a newly improved *LocTextCorpus*. Combined with an automatic named-entity recognizer, *LocText* achieved high precision (P = 86*%*±4). After completing development, we mined the latest research publications for three organisms: human (*Homo sapiens*), budding yeast (*Saccharomyces cerevisiae*), and thale cress (*Arabidopsis thaliana*). Examining 60 novel, text-mined annotations, we found that 65% (human), 85% (yeast), and 80% (cress) were correct. Of all validated annotations, 40% were completely novel, i.e. did neither appear in the annotations nor the text descriptions of Swiss-Prot.

**Conclusions:**

*LocText* provides a cost-effective, semi-automated workflow to assist database curators in identifying novel protein localization annotations. The annotations suggested through text-mining would be verified by experts to guarantee high-quality standards of manually-curated databases such as Swiss-Prot.

**Electronic supplementary material:**

The online version of this article (doi:10.1186/s12859-018-2021-9) contains supplementary material, which is available to authorized users.

## Background

The subcellular location of a protein is an important aspect of its function because the spatial environment constrains the range of operations and processes. For instance, all processing of DNA happens in the nucleus or the mitochondria. In fact, subcellular localization is so important that the Gene Ontology (GO) [1], the standard vocabulary for protein functional annotation, described it by one of its three hierarchies (*Cellular Component*). Many proteins function in different locations. Typically, one of those constitutes the *native* location, i.e. the one in which the protein functions most importantly.

Despite extensive annotation efforts, experimental GO annotations in databases are not nearly complete [2]. Automatic methods may close the annotation gap, i.e. the difference between experimental knowledge and database annotations.

Numerous methods predict location from homology-based inference or sequence-based patterns (sorting signals). These include: *WoLF PSORT* [3], *SignalP* [4], *CELLO* [5], *YLoc* [6], *PSORTb* [7], and *LocTree3* [8]. Text mining-based methods can also “predict” (extract) localization, with the added benefit of linking annotations to the original sources. Curators can compare those resources to validate the suggested annotations and add annotations to high-quality resources such as Swiss-Prot [9] or those for model organisms, e.g. *FlyBase* [10]. An alternative to finding annotations in the free literature is mining controlled texts, such as descriptions and annotation tags in databases [11–13]. Despite numerous past efforts, however, very few text mining systems succeeded in assisting GO curation [14]. A notable exception is *Textpresso* [15], which was integrated into the GO cellular component annotation pipeline of *WormBase* [16] and sped up annotation tenfold over manual curation [17]. Similar computer-assisted curation pipelines have since been implemented for other model organisms [18], but no generic solution for the usage of text mining tools to experts is extensively used yet [19, 20].

Literature-based text mining methods begin with *named-entity recognition* (*NER*), namely the recognition of names of entities, such as proteins or cellular compartments, mentioned within the text. These entities then have to be *normalized*, i.e. disambiguated by mapping the names to exact identifiers in controlled vocabularies (e.g. proteins mapped to UniProtKB [21] and cell compartments to GO). The next task is the *relation extraction* (*RE*) in which relationships between the entities have to be deduced from the semantic context. As an example, in the sentence “CAT2 is localized to the tonoplast in transformed Arabidopsis protoplasts”, PMID (PubMed Identifier) 15377779, the relationship of “CAT2” (UniProtKB: P52569) localized to “tonoplast” (GO:0009705) must be established. Most existing GO annotation methods either coarsely associate all pairs of entities that are co-mentioned in a same sentence or otherwise aggregate the statistics of one or more levels of co-mention (such as the same sentence, paragraph, section, or document). Examples of this include the *CoPub Mapper* [22], *EBIMed* [23], and the *COMPARTMENTS* database [24]. *Textpresso* used manually defined regular expressions. Few methods machine-learned the semantics of text, even if only learning *bags of words* (i.e. disregarding grammar) [25, 26]. Newer methods modeled the syntax of text too (i.e. considering grammar) though were not validated yet in practice for database curation [27–30]. The most recent method of this type [31] probed the discovery of novel protein localizations in unseen publications. However, the method performed poorly in extracting unique relations, i.e. to find out that the same localization relation is described in a publication multiple times but using different synonymous (e.g. due to abbreviations or different spellings). Related to this, the method did not normalize tagged entities; thus, the relations could not be mapped to databases.

To the best of our knowledge, the new method, *LocText*, is the first method to implement a fully-automated pipeline with NER, RE, normalized entities, and linked original sources (necessary for database curation) that machine-learnt the semantics and syntax of scientific text. The system was assessed to achieve high accuracy in a controlled corpus (*intrinsic evaluation*), and to retrieve novel annotations from the literature in a real task (*extrinsic evaluation*).

## Results

### Most relations found in same or consecutive sentences

**Fig. 1:**
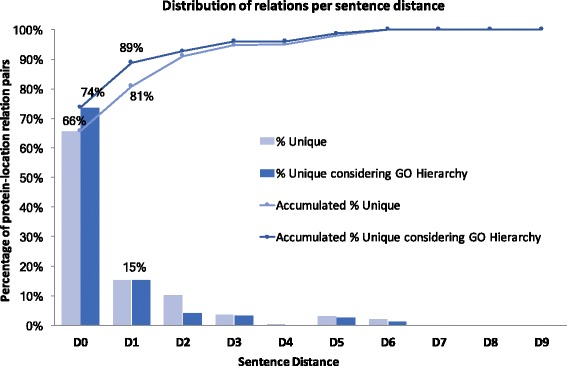
Most related protein and localizations closed to each other. Repetitions of relationships were collapsed at the document level after normalizing the entities: proteins to UniProtKB and localizations to GO. In the *LocTextCorpus*, the majority of unique relations were annotated between entities occurring in the same sentence (distance 0 = D0; 66% of all relations) or in adjacent sentences (dist. 1 = D1; 15%). Combined, D0+D1 accounted for 81% of the relations. Removing repetitions when considering the GO hierarchy (children identifiers are more exact than their parents), D0+D1 accounted for 89% of all unique relationships

The controlled *LocTextCorpus* had annotated 66% of all protein-location unique relations (i.e. collapsing repetitions, “Methods” section) in the same sentence (D0, where *Dn* means that the relation covers entities *n* sentences apart) and 15% in consecutive sentences (D1; Fig. 1). When the GO hierarchy was also considered to collapse redundant relations, D0 (same sentence) increased to 74% (e.g. “lateral plasma membrane”, GO:0016328, overshadowed the less detailed “plasma membrane”, GO:0005886). Consequently, a method that extracted only same-sentence relationships could maximally reach a recall of 74%; at 100% precision, the maximal F-score of such a method would be 85%. Methods that extracted both D0 (same-sentence) and D1 (consecutive sentences) would have a maximal recall of 89% (max. F=94*%*). Considering more distant sentences would rapidly increase the pairs of entities to classify and, with this, likely reduce a method’s precision and substantially increase processing time. *LocTextCorpus* had annotated relationships up to sentence distances of nine (D9). However, after collapsing repeated relations, the maximum distance was six (D6).

### Intrinsic evaluation: relation extraction (RE) and named-entity extraction (NER) succeeded

*LocText* (RE) and *STRING Tagger* (NER) (Methods) independently performed well on the *LocTextCorpus*: *LocText* (RE only) reached P=93*%* at R=68*%* (F=79*%*± 3; Table 1). A high precision was achieved while closely reaching the maximum possible recall for considering only same-sentences relations (D0; max. R=74*%*). The *Baseline* (using manually-annotated entities; Methods) also performed well (P=75*%* at R=74*%*; F=74*%*± 3). A comparative Precision-Recall (PR) curve analysis is shown in Additional file 1: Figure S3. The *STRING Tagger* benchmarked on overlapping normalized entities obtained an aggregated F=81*%*± 1, for the entities Protein (F=79*%*± 2), Location (F=80*%*± 3), and Organism (F=94*%*± 1; Table 1). The precision for the entities Location (P=90*%*) and Organism (P=96*%*) was much higher than for Protein (P=80*%*).

**Table 1:** *LocText* (RE only) and *STRING Tagger* (NER); intrinsic evaluation

Method and evaluation	P	R	F ±*StdErr*
*STRING Tagger* *Total*	84%	78%	81% ± 1
*STRING Tagger* on Protein	80%	78%	79% ± 2
*STRING Tagger* on Location	90%	71%	80% ± 3
*STRING Tagger* on Organism	96%	92%	94% ± 1
*LocText*, with manual entities	93%	68%	79% ± 3
*Baseline*, with manual entities	75%	74%	74% ± 3

Performances of the NER and RE components independently evaluated on the *LocTextCorpus*; P=precision, R=recall, F ±*StdErr*=F-measure with standard error

**Fig. 2:**
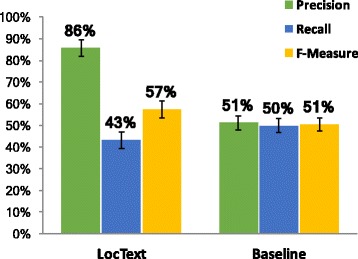
*LocText* full pipeline (NER + RE); intrinsic evaluation. Using the *STRING Tagger*-extracted (“predicted”) entities, both *LocText* and *Baseline* had low and comparable F-measure (F=57% ± 4 and F=51% ± 3, resp.), however *LocText* was optimized for precision (P=86%)

The full *LocText* relation extraction pipeline (NER + RE) achieved high precision (P=86*%*) at the cost of low recall (R=43*%*; F=57*%*± 4, Fig. 2). The *Baseline* (using tagged entities) remained low in precision (P=51*%*) and recall (R=50*%*;*F*=51*%*± 3). Recall might be so low because the errors in RE and NER cumulate: mistakes in identifying the protein, the location, or their relation lead to wrong annotations.

### Extrinsic evaluation: high accuracy enables database curation

Encouraged by the high precision of *LocText*, it was applied to extract protein localization GO annotations from recent PubMed abstracts (*NewDiscoveries_human*, *NewDiscoveries_yeast*, and *NewDiscoveries_cress*; “Methods” section). *LocText* extracted ∼24k unique GO annotations, ∼11k of which (46%) were not found in Swiss-Prot. Some annotations were found in several abstracts. The reliability of the *LocText* annotations increased when found more often. For instance, 10% of the human annotations were found in three or more abstracts (corresponding numbers for yeast: 14%, and thale cress: 6%).

For each organism, the first 20 annotations observed in exactly three abstracts were reviewed. Of the 20 GO annotations for human, 13 (65%) were novel (Table 2; examples of mined novel GO annotations in Additional file 1: Table S2); three of these were more detailed versions of the Swiss-Prot annotations (i.e. child terms in the GO hierarchy). 10 of the 20 had no related annotation in Swiss-Prot (50%). For yeast and cress the novelty fraction was even higher: 85% for yeast (60% without related annotation) and 80% for thale cress (55% without related annotation). The total number of correct novel GO annotations was 46 of 60 (77%) of which 33 (55%) had no related Swiss-Prot annotation.

**Table 2:** *LocText* found novel GO annotations in latest publications; extrinsic evaluation

Org.	#	C	C&NR	C&NT	C&NR,NT
Human	20	13 (65%)	10 (50%)	9 (45%)	7 (35%)
Yest	20	17 (85%)	12 (60%)	6 (30%)	4 (20%)
Cress	20	16 (80%)	11 (55%)	9 (45%)	7 (35%)
*Total*	60	46 (77%)	33 (55%)	24 (40%)	18 (30%)

*LocText* mined protein location relations not tagged in Swiss-Prot in latest publications: 2012-2017 for (column *Org.*=organism) human and 1990-2017 for yeast and cress. (#) 60 novel text-mined annotations (20 for each organism) were manually verified: (*C*=correct) 77% were correct; 55% were correct and had *no relation* (*NR*) in Swiss-Prot; 40% were correct and were *not in text* (*NT*) descriptions of Swiss-Prot; 30% were correct and neither had a relation nor appeared in text descriptions

Upon closer inspection of Swiss-Prot, we found that some of the allegedly novel predictions could have been found in Swiss-Prot text descriptions or other annotations (e.g. biological processes). Still, 9 of the 20 (45%) human annotations were not found (considering also texts) in Swiss-Prot (35% without related annotation in Swiss-Prot considering the GO hierarchy). At that point, we could have gone back and dug deeper, but we could not automate the identification of “find in Swiss-Prot” because the relations were not found through the standard Swiss-Prot tags. The corresponding numbers for yeast and cress were 30% (20% without related annotation) and 45% (35% without related annotation), respectively. The total number of verified completely novel GO annotations not in Swiss-Prot remained as high as 24 out of 60 (40%), of these 18 (30% of 60) had no relation in Swiss-Prot.

23% of the verified predictions were wrong. Half of these errors originated from incorrect proteins, typically due to short and ambiguous abbreviations in the name. For example, “NLS” was wrongly normalized to protein O43175, yet in all texts they referred to “nuclear localization signals”. “FIP3” was wrongly recognized as “NF-kappa-B essential modulator” (Q9Y6K9) while in the three abstracts in which it was found, it referred to “Rab11 family-interacting protein 3” (O75154). The same abbreviation is used for both proteins making this a perfect example how text mining can be beaten by innovative naming. Another 14% of the errors were due to a wrong named-entity localization prediction. For example, in PMID 22101002, the P41180 was correctly identified with the abbreviation CaR, and yet a same abbreviation in the text was also wrongly predicted to be the localization “contractile actomyosin ring”.

The remaining 36% of the errors were due to a wrong relationship extraction. For example, the relation that the protein Cx43 (connexin 43, or “gap junction alpha-1 protein” P17302) is/acts in microtubules could not be fully ascertained from the sentence: “Although it is known that Cx43 hemichannels are transported along microtubules to the plasma membrane, the role of actin in Cx43 forward trafficking is unknown” (PMID 22328533). Another wrongly predicted relationship was OsACBP2 (Q9STP8) to cytosol where the seemingly text proof explicitly negated the relationship: “Interestingly, three small rice ACBP (OsACBP1, OsACBP2 and OsACBP3) are present in the cytosol in comparison to one (AtACBP6) in Arabidopsis” (PMID 26662549). Other wrongly extracted relationships did not show any comprehensible language patterns and were likely predicted for just finding the protein and location co-mentioned.

## Discussion

Achieving high precision might be the most important feature for an automatic method assisting in database curation. Highly-accurate databases such as Swiss-Prot or those of model organisms need to expert-verify all annotations. Focusing on few reliable predictions, expert curators minimize the resources (time) needed to confirm predictions. The manual verification of the 60 GO annotations extracted with *LocText* from recent PubMed abstracts took three person-hours (20 annotations per hour; 60 abstracts per hour). Seventy seven percent of the *LocText* predicted annotations were correct, i.e. an unexperienced expert (we) could easily add ∼120 new annotations on an average 9-5 day to the UniProtKB repository.

The *LocText* method was very fast: it took 45 min to process ∼ 37k PubMed abstracts on a single laptop (MacBook Pro 13-inch, 2013, 2 cores). These ∼ 37k abstracts spanned a wide range of the most recent (from 2012 to 2017) research on human proteins localizations. Twenty one percent of the running time was spent to extract the named entities (*STRING Tagger)*, 26% on text parsing (spaCy), and 52% on pure relationship extraction (*LocText*). If parallelized, *LocText* could process the entire PubMed in near real time.

We discarded relations spanning over more than two sentences (distance ≥1), as the marginal improvements in recall and F-measure did not justify the significant drops in precision. Nevertheless, extracting relations between two neighbor sentences (D1) might increase recall in the future (from 66 to 81% unique relations disregarding the GO hierarchy and 74 to 89% considering the hierarchy).

One important question often neglected in the text mining literature is how well the performance estimates live up to the reality of users, for instance of database curators. Much controversy has followed the recent observations that many if not most published results even in highly-regarded journals (Science and Nature) are not reproducible or false [32–34]. As a curiosity, a GO annotation predicted by *LocText* (deemed wrong upon manual inspection) was found in three journals that were retracted (PMIDs 22504585 and 22504585; the third 23357054 duplicated 22504585). The articles, written by the same authors, were rejected after publication as “expert reviewers agreed that the interpretation of the results was not correct” (PMID 22986443). This work has added particular safe-guards against over-estimating performance (additional data set not used for development), and for gauging performance from the perspective of the user (extrinsic vs. intrinsic evaluation). With all these efforts, it seems clear that novel *GO annotations* suggested by *LocText* have the potential to significantly reduce annotation time (as compared to curators manually searching for new publications and reading those) yet still require further expert verification.

## Conclusions

Here, we presented *LocText*, a new text mining method optimized to assist database curators for the annotation of protein subcellular localizations. *LocText* extracts protein*-in-*location relationships from texts (e.g. PubMed) using syntax information encoded in parse trees. Common language patterns to describe a localization relationship (e.g. “co-localized in”) were learned unsupervised and thus the methodology could extrapolate to other annotation domains.

*LocText* was benchmarked on an improved version of *LocTextCorpus* [35] and compared against a *Baseline* that relates all proteins and locations co-mentioned in a same sentence. Benchmarking only the relation extraction component, i.e. with manually annotated entities, *LocText* and *Baseline* appeared to perform comparably. However, *LocText* achieved much higher precision (P(*LocText)* =93*%* vs. P(*Baseline*) =75*%*). The full pipeline combining the *STRING Tagger* (NER) with *LocText* (RE) reached a low F-measure (F=57*%*± 4) and a low recall (R=43*%*). However, it was optimized for the high precision (P(*LocText*) =86*%* vs. P(*Baseline*) =51*%*).

*LocText* found novel GO annotations in the latest literature for three organisms: human, yeast, and thale cress. 77% of the examined predictions were correct localizations of proteins and were not annotated in Swiss-Prot. More novel annotations could successfully be extracted for yeast and cress (∼80%) than for human (∼65%). Novel annotations that were not traceable from Swiss-Prot (either from annotation tags or from text descriptions) were analyzed separately. Using this definition for *novel annotations*, 40% of all findings were novel. Unexperienced curators (we) validated 20 predicted GO annotations in 1 person-hour. Assisted by the new *LocText* method, curators could enrich UniProtKB with ∼120 novel annotations on a single job day. Advantaging existing automatic methods (*Baseline* with accuracy of 40%-50%), *LocText* could cut curation time in half. Compared to solely manual curation (still common in biological databases), the new method can reduce efforts and resources greatly.

All code, data, and results were open sourced from the start and are available at http://tagtog.net/-corpora/LocText. The new written code added relationship extraction functionality to the *nalaf* framework of natural language processing [36].

## Methods

### Named-entity recognition (NER)

**Fig. 3:**
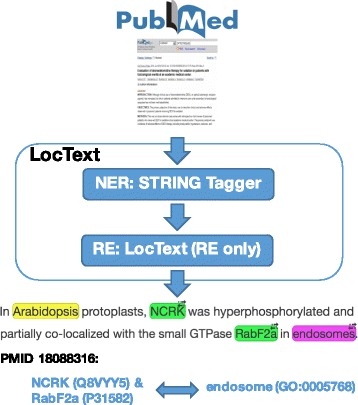
*LocText* pipeline. The input are text documents (e.g. PubMed). First, the *STRING Tagger* recognizes named entities (NER): proteins (green in the example; linked to UniProtKB), cellular localizations (pink; linked to GO), and organisms (yellow; linked to NCBI Taxonomy). Then, the relation extractor (RE) of *LocText* resolves which proteins and localizations are related (as in “localized in”). The output is a list of text-mined relationships (GO annotations) linked to the original text sources (e.g. PMIDs)

The complete *LocText* pipeline consisted of a NER component stacked with a pure RE component (Fig. 3). The RE component was the focus of this work, and its implementation is explained in the following subsections. For NER we reused the existing dictionary-based *STRING Tagger*, which is described in detail in earlier publications [24, 37]. We employed *STRING Tagger* to extract the entities from the text: proteins (more generally, gene or gene products), subcellular localizations, and organisms. Next, we needed to map these to databases, namely to UniProtKB accession numbers, to GO Cellular Component identifiers, and to NCBI Taxonomy identifiers (note: this map is referred to as *normalization* in the text mining community). The method extracts text mentions and the normalized identifiers of entities; it maps proteins to STRING identifiers. We mapped these to UniProtKB accession numbers and ran the Python-wrapped tagger through an in-house Docker-based web server.

The *STRING Tagger* allows the selective usage of organism-dependent dictionaries for protein names. We ran the tagger against the *LocTextCorpus* (see, “Text corpora” section) having selected the dictionaries of human (NCBI Taxonomy: 9606), yeast (NCBI 4932), and thale cress (NCBI 3702). On the sets of documents *NewDiscoveries_human, NewDiscoveries*_*yeast*, and *NewDiscoveries_cress* (Text corpora), we selected only the corresponding organism. We did not consider this selective choice of articles and dictionaries to bias results as this is standard for the curation of model organisms [10, 18, 36]. As another option of the *STRING Tagger*, we also annotated the proteins of other organisms if the protein and organism names were written close to each other in text. For reference, we ran the tagger against *LocTextCorpus* with exact parameters (options): *ids=-22,-3,9606,4932,3702 autodetect=true*. We did not modify the tagger in any way except for removing “Golgi” from the list of *stopwords* (blacklist of names not to annotate) as it likely referred to “Golgi apparatus” in publications known to mention cellular components. We filtered the results by GO identifier to only allow those that were (part of) cell organelles, membranes, or extracellular region. We also explicitly filtered out all tagged cellular components that constituted a “macromolecular complex” (GO:0032991) as in most cases they were enzyme protein complexes, which we did not study (they overlap with the molecular function and biological process hierarchies of the GO ontology). We evaluated the *STRING Tagger* in isolation for NER (“Results” section).

### Relation extraction (RE)

We reduced the problem of relationship extraction to a binary classification: for pairs of entities Prot/Loc (protein/location), decide if they are related (true or false). Several strategies for the generation of candidate pairs are possible, e.g. the enumeration of all combinations from all {Prot/Loc} mentioned in a document. During training, “repeated relation pairs” are used, i.e. the exact text offsets of entities are considered, as opposed to the entity normalizations only (Evaluation). The pairs marked as relations in an annotated corpus (*LocTextCorpus*) are positive instances and other pairs are negative instances. For our new method, we generated only pairs of entities co-occurring in the same sentence. This strategy generated 663 instances (351 positive, 312 negative). Instances were represented as a sentence-based sequence of words along with syntax information (see, Feature selection). We also designed ways to generate and learn from pairs of entities mentioned in consecutive sentences (e.g. the protein mentioned in one sentence and the location in the next). However, we discarded this in the end (“Discussion” section). We modeled the instances with support vector machines (SVMs; [38]). We used the *scikit-learn* implementation with a linear kernel [39, 40]. Neither the tree kernel [41] implemented in SVM-light [42, 43], nor the radial basis function kernel performed better. Other models such as random forests or naive Bayes methods (with either Gaussian, Multinomial, or Bernoulli distributions) also did not perform better in our hands; logistic regression also performed worse, however, within standard error of the best SVM model. For syntactic parsing, we used the python library spaCy (https://spacy.io). For word tokenization, we used our own implementation of the *tmVar*’s tokenizer [36, 44]. This splits contiguous letters and numbers (e.g. “P53” is tokenized as “P” and “53”).

### Feature selection

An instance (positive or negative) is defined as a protein location pair (Prot/Loc) that carries contextual information (the exact text offsets of entities are used). We contemplated features from five different sources: corpus-based, document-based, sentence-based, syntax-based, and domain-specific. The first four were *domain agnostic*. Tens of thousands of features would be generated (compared to 663, the number of instances). Many features, however, were highly correlated. Thus, we applied feature selection. First, we did leave-one-out feature selection, both through manual and automatic inspection (on the validation set, i.e. when cross-training). In the end, by far the most effective feature selection strategy was the Lasso L1 regularization [45]. We ran the *scikit-learnLinearSVC* implementation with penalty = L1 and C = 2 (SVM trade-off hyperparameter). The sparsity property of the L1 norm effectively reduced the number of features to ∼ 300 (ratio of 2 = num. instances / num. features). We applied independent feature selection whether we used the manually annotated entities or the entities identified by *STRINGTagger*. Both yielded almost equal features. Ultimately, we only used the following five feature types.

*Entity counts in the sentence (domain agnostic, 2 features):* individual entity counts (for protein, location, and organisms too) and the total sum. Counts were scaled to floats [0, 1] dividing them by the largest number found in the training data (independently for each feature). If the test data had a larger number than previously found while training, its scaled float would be bigger than 1 (e.g. if the largest number in training was 10, a count of 11 in testing would be scaled to 1.1).

*Is protein a marker (domain specific, 1 feature):* for example, green fluorescent protein (GFP), or red fluorescent protein (RFP). This might be a problem of the *LocTextCorpus* guidelines. Nonetheless, disregarding protein markers seems a reasonable step to curate databases.

*Is the relation found in Swiss-Prot (domain specific, 1 feature):* we leveraged the existing annotations from Swiss-Prot.

*N-grams between entities in linear dependency (domain agonistic, 57% of ∼ 300 features)*: the n-grams (*n* = 1, 2, or 3*)* of tokens in the linear sentence between the pair of entities Prot and Loc. The tokens were mapped in two ways: 1) word lemmas in lower case masking numbers as the special *NUM* symbol and masking tokens of mentioned entities as their class identifier (i.e. *PROTEIN, LOCATION, or ORGANISM)*; 2) words part of speech (POS). In a 2- or 3-gram, the entity on the left was masked as *SOURCE* and the end entity on the right as *TARGET*.

*N-grams of syntactic dependency tree(domain agnostic, 42% of ∼ 300 features)*: the shortest path in the dependency parse tree connecting Prot and Loc was computed (Additional file 1: Figure S1). The connecting tokens were mapped in three ways: 1) word lemmas with same masking as before; 2) part of speech, same masking; 3) syntactic dependencies edges (e.g. *preposition* or *direct object*). Again, we masked the pair of entities in the path as *SOURCE* and *TARGET*. The direction of the edges in the dependency tree (going up to the sentence root or down from it) was not outputted after feature selection.

The representation of the sentences as dependency graphs was inspired by Björne’s method for event extraction in BioNLP’09 [46]. The n-gram features, both linear- and dependency-tree-based, that were ultimately chosen after unsupervised feature selection yielded comprehensible language patterns (Additional file 1: Table S1). In the Supplementary Online Material (SOM), we listed all the features that were finally selected (Additional file 1: Figure S2).

### Evaluation

High performance of a method in a controlled setting (*intrinsic evaluation*) does not directly translate into high performance in a real task (*extrinsic* evaluation) [47]. To address this, we evaluated the new *LocText* method in both scenarios, namely, in a well-controlled corpus using standard performance measures and in the real setting of extracting novel protein localizations from the literature. Either way, and always with database curation in mind, we asked: given a scientific text (e.g. PubMed article), what protein location relationships does it attest to? For instance, a publication may reveal “Protein S” (UniProtKB: P07225) to function in the “plasma membrane” (GO:0005886). To extract this relation, it is indifferent under which names the protein and location are mentioned. For instance, P07225 can also be named “Vitamin K-dependent protein S” or “PROS1” or abbreviated “PS” and GO:0005886 can also be called “cell membrane” or "cytoplasmic membrane” or abbreviated “PM”. Further, it does not matter if the relation is expressed with different but semantically equivalent phrases (e.g. “PROS1 was localized in PM” or “PM is the final destination of PROS1”). Regardless of synonymous names and different wordings, repeated attestations of the relation within the same document are all the same. In other words, we evaluated relationship extraction at the document level and for normalized entities.

In intrinsic evaluation, the annotated relations of a corpus were grouped by document and represented as a unique set of normalized entity pairs of the form (Prot=protein, Loc=location), e.g. (P07225, GO:0005886). A tested known relationship (Prot_test_, Loc_test_) was considered as correctly extracted (*true positive* = tp), if at least one text-mined relation (Prot_pred_*,* Loc_pred_) matched it, with both Prot and Loc correctly normalized: 1) Prot_test_ and Prot_pred_ must be equal or have a percentage sequence identity 90% (to account for cases where likely a same protein entries can have multiple identifiers in UniProtKB/TrEMBL [48]); and 2) Loc_test_ and Loc_pred_ must be equal or Loc_pred_ must be a leave or child of Loc_test_ (to account for the tree-based GO hierarchy). For example, a tested (P07225, GO:0005886) relation and a predicted (P07225, GO:0016328) relation correctly match: the proteins are the same and GO:0016328 (“lateral plasma membrane”) is a part of and thus more detailed than GO:0005886 (“plasma membrane”). Any other predicted relationship was wrong (*false positive* = fp), and any missed known relationship was also punished (*false negative* = fn). We then computed the standard performance measures for *precision*$\left (\text {P}= \frac {tp}{tp + fp}\right)$, *recall*$\left (\text {R}= \frac {tp}{tp + fn}\right)$, and *F-measure*$\left (\text {F}= 2 * \frac {P * R}{P + R}\right)$ (all three multiplied by 100, in percentages).

We evaluated relationship extraction in isolation (using manually-annotated entities, i.e. the proteins and localizations) and as a whole (with predicted entities). Given the importance of the NER module (wrongly predicted entities lead to wrongly predicted relationships), we also evaluated the NER in isolation. We considered a predicted named entity as successfully extracted (*tp*) if and only if its text offsets (character positions in a text-string) *overlapped* those of a known entity and its normalized identifier matched the same test entity’s normalization (also accounting for similar proteins and for the GO hierarchy). Any other predicted entity was counted as *fp* and any missed entity as *fn*. In analogy, we computed P, R, and F for named-entity recognition.

We evaluated methods in 5-fold cross-validation with three separate sets as follows. First, we split a fold into the three sets by randomizing the publications; this lessens redundancy as different publications mention different localizations. Sixty percent of documents served to train (train set), 20% to cross-train (validation set), i.e. to optimize parameters such as in feature or model selection. The remaining 20% were used for testing (test set). The performance on the test set was compiled only after all development had been completed and was thus not used for any optimization. Finally, we repeated the folds four more times, such that each article had been used for testing exactly once. We computed the standard error (*StdErr*) by randomly selecting 15% of the test data without replacement in 1000 (*n*) bootstrap samples. With 〈*x*〉 as the overall performance for the entire test set and *x*_*i*_ for subset *i*, we computed:

1 $$\begin{array}{*{20}l} \sigma = \sqrt{\frac{1}{n-1} \sum\limits_{i=1}^{n} (x_{i} - \langle x \rangle)^{2}} && StdErr = \frac{\sigma}{\sqrt{n}} \end{array} $$

In extrinsic evaluation, the complete *LocText* pipeline (i.e. NER + RE) extracted from large sets of unannotated PubMed abstracts novel protein localizations (namely, GO annotations not tagged in Swiss-Prot). A unique protein-location relation could be found in one or more documents. The assumption is: the more document hits, the more reliable the extracted relation. For a number of extracted unique relations, one person manually reviewed the originating and linked documents. For each “predicted” relation, we stopped our analysis when we found proof of the annotation. We deemed the prediction to be wrong if we found no textual proof in the abstracts.

### Text corpora

To train and formally benchmark the new method (intrinsic evaluation), we had only access to a custom-built corpus, for simplicity referred to as *LocTextCorpus* [35]. We could not reuse other annotated corpora as they did not provide annotations at the text level or had incompatible annotations. Specifically, the *BioNLP’09* corpus [28] and the *BC4GO* corpus [49] appeared very promising but contained particular features that made it impossible for us to use those valuable resources. *BioNLP’09*, for instance, annotated *events* (relationships) not requiring the textual mention of the protein or localization entities, some location mentions contained extraneous words that were part of the phrase but not strictly part of the location names, and some locations were not only subcellular localizations but specific cells or body tissues. *BC4GO* contained neither exact text-level annotations of the entities nor the relationships.

We had previously annotated the *LocTextCorpus* with the *tagtog* tool [50]. For this work, we added 8 missing protein normalizations. *LocTextCorpus* collected 100 abstracts (50 abstracts for human proteins, 25 for yeast, and 25 for thale cress) with 1393 annotated proteins, 558 localizations, and 277 organisms. The organism annotation had been crucial to correctly map the protein sequence, e.g. to distinguish the human *Protein S* (P07225/PROS_HUMAN) from its mouse ortholog (Q08761/PROS_MOUSE). The corpus annotated 1345 relationships (550 protein-localization + 795 protein-organism). When removing repeated relations through entity normalization (Evaluation), the number of unique protein-localization relations was 303. Relationships of entities mentioned in any sentence apart had been annotated (Results). That is, the related protein and location entities could have been mentioned in the same sentence (sentence distance=0, D0), or contiguous sentences (sentence distance=1, D1), or farther away (D ≥ 2). The agreement (F-measure) between two annotators (an estimation of the quality of annotations) reached as high as: F=96 for protein annotation, F=88 for localization annotation, and F=80 for protein-localization relationship annotation. *LocTextCorpus* was used to train, select features, and test (in cross-validation) the new *LocText* method.

Furthermore, and to assess how the new method *LocText* could assist in database curation in practice, three sets of PubMed abstracts were added: *NewDiscoveries_human*, *NewDiscoveries_yeast*, *NewDiscoveries_cress*. For each organism, keyword searches on PubMed revealed recent publications that likely evidenced (mentioned) the localization of proteins (e.g. the search for human *http://bit.ly/2nLiRCK*). The search for all human-related journals published between 2012 to 2017/03 yielded ∼ 37k documents (exactly 37454). For publication years from 1990 to 2017/03, the search obtained ∼ 18k (17544) documents for yeast and ∼ 8k (7648) for cress. These documents were not fully tagged. They were only used for final *extrinsic* evaluation, and only after the method had been finalized. In other words, those abstracts never entered any aspect of the development/training phase.

### Existing methods for comparison

Two previous methods that used machine learning techniques to model syntax also extracted protein localization relationships [27, 31]. However, neither methods were made available. We found no other machine learning-based methods available for comparison. The *Textpresso* system uses regular expressions and is used in database curation [15]. The method, however, is packaged as a search index (suited to their specialized corpora, e.g. for WormBase) and not as an extraction method. We were not able to run it for new corpora.

Other methods exist that follow a simple heuristic: if two entities are *co-mentioned* then they are related [22–24]. The heuristic of same-sentence co-occurrence (as opposed to e.g. document co-occurrence) is simple and yields top results. Therefore, this was considered as the *Baseline* to compare the new method against.

## Additional file

Additional file 1 Supporting online material. PDF document with supplemental figures and tables (Fig. S1-S3, Tables S1-S2), one per page. (PDF 238 kb)

## Abbreviations

F: F-measure
GO: Gene ontology
Loc: Location
NER: Named-entity recognition
P: Precision
Prot: Protein
R: Recall
RE: Relation extraction
