# Supplementary material for: LocText: relation extraction of protein localizations to assist database curation
Source: BMC Bioinformatics. 2018 Jan 17;19:15. doi: 10.1186/s12859-018-2021-9 (PMC5773052; doi:10.1186/s12859-018-2021-9)

Supporting online material
for:

LocText: relation extraction of protein localizations to assist database curation

Juan Miguel Cejuela, Shrikant Vinchurkar, Tatyana Goldberg, Madhukar Sollepura Prabhu Shankar, Ashish Baghudana, Aleksandar Bojchevski, Carsten Uhlig, André Ofner, Pandu Raharja-Liu, Lars Juhl Jensen, and Burkhard Rost

1. Short description of Supporting Online Material

Some results not shown in main paper but supporting some described findings.

2. Material

(starts in next page; one Table/Figure per page)

Fig. S1. Parse Tree Features. *LocText* derives features from parsed syntax trees: dependencies between tokens (e.g. "to" prepositions "the trans-Golgi network"), noun phrases (e.g. the AP-1 μ adaptin subunit"), part of speech tags (e.g. "localized" is a verb, past participle), linear distance between tokens (e.g. "adaptin" and "Golgi" are 10-tokens away from each other), or dependency distances between tokens (e.g. "adapting" and "Golgi" are 4-dependencies away).

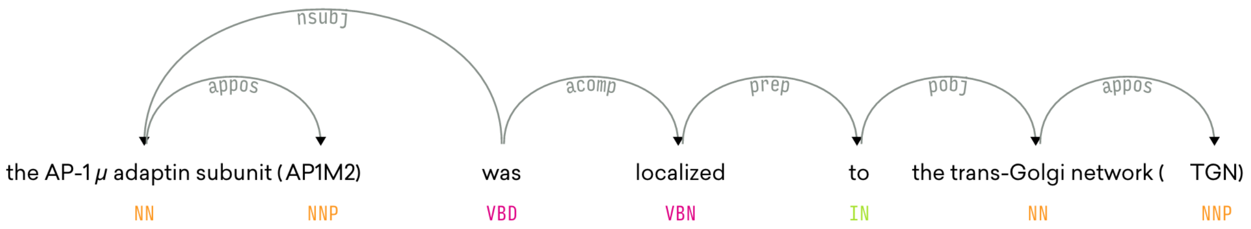

Table S1. Sample features after L1 regularization selection.

Feature name	Explanation
Total count of entities	Float (scaled [0, 1]), count of entities (proteins + localizations + organisms) in sentence-based instance.
Is Protein Marker	(Binary) test whether the marked protein text was equal to a list of manually defined protein markers: GFP, CYH2, ALG2, MSB2, KSS1, KRE11, SER2.
PROTEIN localize	Binary, Linear Dependency (LD) feature. Test if the sentence-based instance contains two consecutive tokens (2-gram), where the first token is part of an protein name and the following has lemma “localize” (e.g. localized).
SOURCE localization at	Binary, LD. Test if the first entity (source) in the sentence-based instance (either protein or location; likely in this case, a protein), is followed by the lemmas “localization” and “at”.
SOURCE proliferation *TARGET*	Binary, Parsing Dependency (PD). Test if the source entity is connected to the second entity (target) by the lemma “proliferation” in the dependency parse tree.
in the *TARGET*	Binary, LD. Test if the lemmas “in” and “the” follow the target entity (likely a location).
SOURCE VERB DET	Binary, LD. Test if the source entity is followed by two tokens, the first a VERB, the next a determiner (DET)
VERB NOUN TARGET	Binary, PD. Test if, in the dependency parse tree, a VERB token connects a NOUN token that connects the target entity.
NSUBJ DOBJ PREP	Binary, PD. Test if, in the dependency parse tree, three tokens are connected with the dependencies NSUBJ (nominal subject) to (direct object) to PREP (prepositional modifier).

Table S2. Examples of novel GO annotations text-mined by *LocText*.

Protein	Localization	Text Source
P61586 human	GO:0005634 (nucleus)	Recent studies have revealed the localization of **RhoA** protein in the cell **nucle-us**, in addition to its distribution in the cytosol and cell membrane. *PMID 26622605, 2015*
P09936 human	GO:0008021 (synaptic vesicle)	Both **synaptic vesicle markers** co-localized with the neuronal marker **PGP 9.5** and exhibited granular accumulation patterns in the **human** and rat ENS. *PMID 24025431, 2013*
P56817 hu-man	GO:0005764 (lysosome)	Here, we report that lack of ubiquitination at Lys-501 (BACE1K501R) does not affect the rate of endocytosis but produces **BACE1** stabilization and accumula-tion of **BACE1** in early and late endosomes/**lysosomes** as well as at the cell membrane. *PMID 23109336, 2012*
P01149 yeast	GO:0005773 (vacuole)	[...] we provided evidence for the existence of an endocytic intermediate(s) from the **yeast Saccharomyces cerevisiae** that is responsible for the transport of the **pheromone alpha-factor** from the plasma membrane to the **vacuole**. PMID 8314797, 1993
P13134 yeast	GO:0005768 (endo-some)	However, **Kex2** localization is not static, and its itinerary apparently involves transiting out of the late Golgi and cycling back from post-Golgi **endosomal** compartments during its lifetime. We tested whether the endocytic pathway could deliver small molecules to **Kex2** from the extracellular medium. Here we report that intramolecularly quenched fluorogenic substrates taken up into intact **yeast** revealed fluorescence due to specific cleavage by **Kex2** protease in **endosomal** compartments. *PMID 10393104, 1999*
Q9FE59 cress	GO:0009705 (plant-type vacuole membrane)	We demonstrate that this motif can reroute other proteins, such as INT4, **SU-CROSE TRANSPORTER2 (SUC2)**, or SWEET1, to the **tonoplast** and that the position of the motif relative to the transmembrane helix is critical. *PMID 22253225, 2012*
O82533 cress	GO:0005829 (cytosol)	Here, we report the identification of a **second nuclear-encoded FtsZ-type protein** from **Arabidopsis** that does not contain a chloroplast targeting se-quence or other obvious sorting signals and is not imported into isolated chloroplasts, which strongly suggests that it is localized in the **cytosol**. *PMID 9836740, 1998*

Fig. S2. List of all finally selected features. Python-readable list of descriptive feature names.

```
[
    "SentenceFeatureGenerator::1.1_counts_individual_int_individual_e_1_[0]",  # 0
    "SentenceFeatureGenerator::1.1_counts_individual_int_individual_e_3_[0]",  # 1
    "SentenceFeatureGenerator::3_order_[0]",  # 2
    "DependencyFeatureGenerator::19_LD_pos_N_gram_LD_1_<NOUN>_[0]",  # 3
    "DependencyFeatureGenerator::19_LD_pos_N_gram_LD_2_<[SOURCE ~~ PUNCT>_[0]",  # 4
    "DependencyFeatureGenerator::19_LD_pos_N_gram_LD_2_<PUNCT ~~ VERB>_[0]",  # 5
    "DependencyFeatureGenerator::19_LD_pos_N_gram_LD_2_<NOUN ~~ NOUN>_[0]",  # 6
    "DependencyFeatureGenerator::19_LD_pos_N_gram_LD_3_<VERB ~~ NOUN ~~ PUNCT>_[0]",  # 7
    "DependencyFeatureGenerator::19_LD_pos_N_gram_LD_3_<NOUN ~~ PUNCT ~~ ADJ>_[0]",  # 8
    "DependencyFeatureGenerator::19_LD_pos_N_gram_LD_3_<NOUN ~~ PUNCT ~~ NOUN>_[0]",  # 9
    "DependencyFeatureGenerator::19_LD_pos_N_gram_LD_3_<PUNCT ~~ NOUN ~~ NOUN>_[0]",  # 10
    "DependencyFeatureGenerator::18_LD_bow_N_gram_LD_1_<as>_[0]",  # 11
    "DependencyFeatureGenerator::18_LD_bow_N_gram_LD_1_<an>_[0]",  # 12
    "DependencyFeatureGenerator::19_LD_pos_N_gram_LD_1_<ADP>_[0]",  # 13
    "DependencyFeatureGenerator::19_LD_pos_N_gram_LD_1_<NUM>_[0]",  # 14
    "DependencyFeatureGenerator::19_LD_pos_N_gram_LD_1_<DET>_[0]",  # 15
    "DependencyFeatureGenerator::19_LD_pos_N_gram_LD_2_<[SOURCE ~~ NOUN>_[0]",  # 16
    "DependencyFeatureGenerator::19_LD_pos_N_gram_LD_2_<PUNCT ~~ DET>_[0]",  # 17
    "DependencyFeatureGenerator::23_PD_pos_N_gram_PD_1_<VERB>_[0]",  # 18
    "DependencyFeatureGenerator::23_PD_pos_N_gram_PD_1_<NOUN>_[0]",  # 19
    "DependencyFeatureGenerator::26_PD_undirected_edges_N_gram_PD_1_<nsubj>_[0]",  # 20
    "DependencyFeatureGenerator::26_PD_undirected_edges_N_gram_PD_1_<prep>_[0]",  # 21
    "DependencyFeatureGenerator::23_PD_pos_N_gram_PD_2_<NOUN ~~ NOUN>_[0]",  # 22
    "DependencyFeatureGenerator::23_PD_pos_N_gram_PD_3_<[SOURCE ~~ VERB ~~ ADP>_[0]",  # 23
    "DependencyFeatureGenerator::23_PD_pos_N_gram_PD_3_<VERB ~~ ADP ~~ NOUN>_[0]",  # 24
    "DependencyFeatureGenerator::23_PD_pos_N_gram_PD_3_<ADP ~~ NOUN ~~ NOUN>_[0]",  # 25
    "DependencyFeatureGenerator::26_PD_undirected_edges_N_gram_PD_3_<prep ~~ pobj ~~ appos>_[0]",  #
26
    "DependencyFeatureGenerator::19_LD_pos_N_gram_LD_1_<PROPN>_[0]",  # 27
    "DependencyFeatureGenerator::18_LD_bow_N_gram_LD_2_<e_1 ~~ e_1>_[0]",  # 28
    "DependencyFeatureGenerator::19_LD_pos_N_gram_LD_2_<DET ~~ ADJ>_[0]",  # 29
    "DependencyFeatureGenerator::19_LD_pos_N_gram_LD_2_<NOUN ~~ PROPN>_[0]",  # 30
    "DependencyFeatureGenerator::19_LD_pos_N_gram_LD_2_<PUNCT ~~ CONJ>_[0]",  # 31
    "DependencyFeatureGenerator::19_LD_pos_N_gram_LD_3_<PUNCT ~~ CONJ ~~ DET>_[0]",  # 32
    "DependencyFeatureGenerator::19_LD_pos_N_gram_LD_3_<CONJ ~~ DET ~~ TARGET]>_[0]",  # 33
    "DependencyFeatureGenerator::19_LD_pos_N_gram_LD_2_<[SOURCE ~~ VERB>_[0]",  # 34
    "DependencyFeatureGenerator::19_LD_pos_N_gram_LD_3_<NOUN ~~ PUNCT ~~ TARGET]>_[0]",  # 35
    "DependencyFeatureGenerator::26_PD_undirected_edges_N_gram_PD_2_<amod ~~ appos>_[0]",  # 36
    "DependencyFeatureGenerator::23_PD_pos_N_gram_PD_3_<[SOURCE ~~ NOUN ~~ TARGET]>_[0]",  # 37
    "DependencyFeatureGenerator::22_PD_bow_N_gram_PD_3_<[SOURCE ~~ form ~~ form>_[0]",  # 38
    "DependencyFeatureGenerator::18_LD_bow_N_gram_LD_1_<,>_[0]",  # 39
    "DependencyFeatureGenerator::18_LD_bow_N_gram_LD_2_<[SOURCE ~~ ,>_[0]",  # 40
    "DependencyFeatureGenerator::18_LD_bow_N_gram_LD_2_<, ~~ TARGET]>_[0]",  # 41
    "DependencyFeatureGenerator::22_PD_bow_N_gram_PD_1_<to>_[0]",  # 42
    "DependencyFeatureGenerator::22_PD_bow_N_gram_PD_1_<exit>_[0]",  # 43
    "DependencyFeatureGenerator::26_PD_undirected_edges_N_gram_PD_1_<advcl>_[0]",  # 44
    "DependencyFeatureGenerator::23_PD_pos_N_gram_PD_2_<VERB ~~ VERB>_[0]",  # 45
    "DependencyFeatureGenerator::26_PD_undirected_edges_N_gram_PD_2_<pobj ~~ prep>_[0]",  # 46
    "DependencyFeatureGenerator::26_PD_undirected_edges_N_gram_PD_2_<prep ~~ advcl>_[0]",  # 47
    "DependencyFeatureGenerator::18_LD_bow_N_gram_LD_1_<in>_[0]",  # 48
    "DependencyFeatureGenerator::19_LD_pos_N_gram_LD_2_<ADP ~~ NOUN>_[0]",  # 49
    "DependencyFeatureGenerator::19_LD_pos_N_gram_LD_2_<ADP ~~ ADJ>_[0]",  # 50
    "DependencyFeatureGenerator::19_LD_pos_N_gram_LD_3_<[SOURCE ~~ VERB ~~ VERB>_[0]",  # 51
    "DependencyFeatureGenerator::19_LD_pos_N_gram_LD_3_<VERB ~~ VERB ~~ ADP>_[0]",  # 52
    "DependencyFeatureGenerator::26_PD_undirected_edges_N_gram_PD_1_<dobj>_[0]",  # 53
    "DependencyFeatureGenerator::26_PD_undirected_edges_N_gram_PD_2_<nsubj ~~ advcl>_[0]",  # 54
    "DependencyFeatureGenerator::26_PD_undirected_edges_N_gram_PD_2_<dobj ~~ amod>_[0]",  # 55
    "DependencyFeatureGenerator::23_PD_pos_N_gram_PD_3_<VERB ~~ NOUN ~~ TARGET]>_[0]",  # 56
    "DependencyFeatureGenerator::18_LD_bow_N_gram_LD_1_<mutant>_[0]",  # 57
    "DependencyFeatureGenerator::19_LD_pos_N_gram_LD_1_<SYM>_[0]",  # 58
    "DependencyFeatureGenerator::18_LD_bow_N_gram_LD_2_<, ~~ the>_[0]",  # 59
    "DependencyFeatureGenerator::19_LD_pos_N_gram_LD_2_<DET ~~ PROPN>_[0]",  # 60
    "DependencyFeatureGenerator::18_LD_bow_N_gram_LD_3_<the ~~ e_1 ~~ e_1>_[0]",  # 61
```

```
    "DependencyFeatureGenerator::22_PD_bow_N_gram_PD_1_<accumulate>_[0]",  # 62
    "DependencyFeatureGenerator::23_PD_pos_N_gram_PD_2_<VERB ~~ ADJ>_[0]",  # 63
    "DependencyFeatureGenerator::23_PD_pos_N_gram_PD_2_<ADP ~~ TARGET]>_[0]",  # 64
    "DependencyFeatureGenerator::26_PD_undirected_edges_N_gram_PD_2_<acl ~~ prep>_[0]",  # 65
    "DependencyFeatureGenerator::23_PD_pos_N_gram_PD_3_<[SOURCE ~~ NOUN ~~ ADP>_[0]",  # 66
    "DependencyFeatureGenerator::23_PD_pos_N_gram_PD_3_<NOUN ~~ ADP ~~ NOUN>_[0]",  # 67
    "DependencyFeatureGenerator::23_PD_pos_N_gram_PD_3_<ADP ~~ NOUN ~~ ADP>_[0]",  # 68
    "DependencyFeatureGenerator::23_PD_pos_N_gram_PD_3_<NOUN ~~ ADP ~~ VERB>_[0]",  # 69
    "DependencyFeatureGenerator::26_PD_undirected_edges_N_gram_PD_3_<pobj ~~ prep ~~ pobj>_[0]",  #
70
    "DependencyFeatureGenerator::26_PD_undirected_edges_N_gram_PD_3_<acl ~~ prep ~~ pobj>_[0]",  # 71
    "DependencyFeatureGenerator::18_LD_bow_N_gram_LD_1_<cell>_[0]",  # 72
    "DependencyFeatureGenerator::18_LD_bow_N_gram_LD_1_<with>_[0]",  # 73
    "DependencyFeatureGenerator::18_LD_bow_N_gram_LD_2_<the ~~ e_2>_[0]",  # 74
    "DependencyFeatureGenerator::18_LD_bow_N_gram_LD_2_<e_2 ~~ of>_[0]",  # 75
    "DependencyFeatureGenerator::18_LD_bow_N_gram_LD_2_<e_1 ~~ and>_[0]",  # 76
    "DependencyFeatureGenerator::18_LD_bow_N_gram_LD_2_<with ~~ the>_[0]",  # 77
    "DependencyFeatureGenerator::19_LD_pos_N_gram_LD_3_<NOUN ~~ ADP ~~ NOUN>_[0]",  # 78
    "DependencyFeatureGenerator::19_LD_pos_N_gram_LD_3_<ADP ~~ NOUN ~~ VERB>_[0]",  # 79
    "DependencyFeatureGenerator::19_LD_pos_N_gram_LD_3_<DET ~~ PROPN ~~ NOUN>_[0]",  # 80
    "DependencyFeatureGenerator::19_LD_pos_N_gram_LD_3_<NOUN ~~ ADP ~~ DET>_[0]",  # 81
    "DependencyFeatureGenerator::26_PD_undirected_edges_N_gram_PD_1_<nsubjpass>_[0]",  # 82
    "DependencyFeatureGenerator::19_LD_pos_N_gram_LD_3_<VERB ~~ DET ~~ TARGET]>_[0]",  # 83
    "DependencyFeatureGenerator::22_PD_bow_N_gram_PD_1_<of>_[0]",  # 84
    "DependencyFeatureGenerator::22_PD_bow_N_gram_PD_1_<cell>_[0]",  # 85
    "DependencyFeatureGenerator::22_PD_bow_N_gram_PD_2_<[SOURCE ~~ of>_[0]",  # 86
    "DependencyFeatureGenerator::23_PD_pos_N_gram_PD_2_<NOUN ~~ VERB>_[0]",  # 87
    "DependencyFeatureGenerator::26_PD_undirected_edges_N_gram_PD_3_<prep ~~ pobj ~~ acl>_[0]",  # 88
    "DependencyFeatureGenerator::26_PD_undirected_edges_N_gram_PD_2_<dobj ~~ acl>_[0]",  # 89
    "DependencyFeatureGenerator::26_PD_undirected_edges_N_gram_PD_2_<acl ~~ pobj>_[0]",  # 90
    "DependencyFeatureGenerator::26_PD_undirected_edges_N_gram_PD_2_<amod ~~ prep>_[0]",  # 91
    "DependencyFeatureGenerator::26_PD_undirected_edges_N_gram_PD_3_<amod ~~ prep ~~ pobj>_[0]",  #
92
    "DependencyFeatureGenerator::22_PD_bow_N_gram_PD_2_<[SOURCE ~~ in>_[0]",  # 93
    "DependencyFeatureGenerator::26_PD_undirected_edges_N_gram_PD_2_<pobj ~~ compound>_[0]",  # 94
    "DependencyFeatureGenerator::18_LD_bow_N_gram_LD_1_<be>_[0]",  # 95
    "DependencyFeatureGenerator::18_LD_bow_N_gram_LD_1_<class>_[0]",  # 96
    "DependencyFeatureGenerator::18_LD_bow_N_gram_LD_1_<relate>_[0]",  # 97
    "DependencyFeatureGenerator::18_LD_bow_N_gram_LD_2_<on ~~ TARGET]>_[0]",  # 98
    "DependencyFeatureGenerator::19_LD_pos_N_gram_LD_2_<ADP ~~ TARGET]>_[0]",  # 99
    "DependencyFeatureGenerator::19_LD_pos_N_gram_LD_3_<[SOURCE ~~ VERB ~~ DET>_[0]",  # 100
    "DependencyFeatureGenerator::22_PD_bow_N_gram_PD_1_<be>_[0]",  # 101
    "DependencyFeatureGenerator::23_PD_pos_N_gram_PD_3_<NOUN ~~ NOUN ~~ VERB>_[0]",  # 102
    "DependencyFeatureGenerator::19_LD_pos_N_gram_LD_2_<NOUN ~~ CONJ>_[0]",  # 103
    "DependencyFeatureGenerator::19_LD_pos_N_gram_LD_2_<CONJ ~~ VERB>_[0]",  # 104
    "DependencyFeatureGenerator::19_LD_pos_N_gram_LD_3_<DET ~~ NOUN ~~ PUNCT>_[0]",  # 105
    "DependencyFeatureGenerator::19_LD_pos_N_gram_LD_3_<CONJ ~~ VERB ~~ VERB>_[0]",  # 106
    "DependencyFeatureGenerator::19_LD_pos_N_gram_LD_3_<VERB ~~ ADP ~~ ADJ>_[0]",  # 107
    "DependencyFeatureGenerator::26_PD_undirected_edges_N_gram_PD_1_<nummod>_[0]",  # 108
    "DependencyFeatureGenerator::23_PD_pos_N_gram_PD_3_<VERB ~~ VERB ~~ ADP>_[0]",  # 109
    "DependencyFeatureGenerator::18_LD_bow_N_gram_LD_1_<expression>_[0]",  # 110
    "DependencyFeatureGenerator::19_LD_pos_N_gram_LD_3_<NOUN ~~ ADP ~~ TARGET]>_[0]",  # 111
    "DependencyFeatureGenerator::18_LD_bow_N_gram_LD_2_<) ~~ be>_[0]",  # 112
    "DependencyFeatureGenerator::19_LD_pos_N_gram_LD_2_<PROPN ~~ PUNCT>_[0]",  # 113
    "DependencyFeatureGenerator::19_LD_pos_N_gram_LD_3_<VERB ~~ ADJ ~~ NOUN>_[0]",  # 114
    "DependencyFeatureGenerator::19_LD_pos_N_gram_LD_3_<NOUN ~~ ADP ~~ VERB>_[0]",  # 115
    "DependencyFeatureGenerator::22_PD_bow_N_gram_PD_1_<from>_[0]",  # 116
    "DependencyFeatureGenerator::26_PD_undirected_edges_N_gram_PD_1_<acomp>_[0]",  # 117
    "DependencyFeatureGenerator::26_PD_undirected_edges_N_gram_PD_1_<advmod>_[0]",  # 118
    "DependencyFeatureGenerator::26_PD_undirected_edges_N_gram_PD_2_<pcomp ~~ prep>_[0]",  # 119
    "DependencyFeatureGenerator::26_PD_undirected_edges_N_gram_PD_3_<nsubj ~~ acomp ~~ prep>_[0]",  #
120
    "DependencyFeatureGenerator::18_LD_bow_N_gram_LD_2_<with ~~ e_1>_[0]",  # 121
    "DependencyFeatureGenerator::19_LD_pos_N_gram_LD_2_<ADP ~~ PROPN>_[0]",  # 122
    "DependencyFeatureGenerator::19_LD_pos_N_gram_LD_2_<PROPN ~~ ADP>_[0]",  # 123
    "DependencyFeatureGenerator::19_LD_pos_N_gram_LD_2_<VERB ~~ PROPN>_[0]",  # 124
```

```
    "DependencyFeatureGenerator::18_LD_bow_N_gram_LD_3_<[SOURCE ~~ colocalizes ~~ with>_[0]",  # 125
    "DependencyFeatureGenerator::19_LD_pos_N_gram_LD_3_<NUM ~~ ADP ~~ DET>_[0]",  # 126
    "DependencyFeatureGenerator::19_LD_pos_N_gram_LD_3_<DET ~~ NOUN ~~ TARGET]>_[0]",  # 127
    "DependencyFeatureGenerator::18_LD_bow_N_gram_LD_3_<[SOURCE ~~ and ~~ e_1>_[0]",  # 128
    "DependencyFeatureGenerator::23_PD_pos_N_gram_PD_2_<[SOURCE ~~ PROPN>_[0]",  # 129
    "DependencyFeatureGenerator::26_PD_undirected_edges_N_gram_PD_2_<conj ~~ pobj>_[0]",  # 130
    "DependencyFeatureGenerator::26_PD_undirected_edges_N_gram_PD_2_<conj ~~ conj>_[0]",  # 131
    "DependencyFeatureGenerator::18_LD_bow_N_gram_LD_2_<[SOURCE ~~ in>_[0]",  # 132
    "DependencyFeatureGenerator::19_LD_pos_N_gram_LD_3_<[SOURCE ~~ NOUN ~~ TARGET]>_[0]",  # 133
    "DependencyFeatureGenerator::18_LD_bow_N_gram_LD_1_<transporter>_[0]",  # 134
    "DependencyFeatureGenerator::18_LD_bow_N_gram_LD_1_<that>_[0]",  # 135
    "DependencyFeatureGenerator::18_LD_bow_N_gram_LD_2_<e_1 ~~ ,>_[0]",  # 136
    "DependencyFeatureGenerator::19_LD_pos_N_gram_LD_2_<ADV ~~ VERB>_[0]",  # 137
    "DependencyFeatureGenerator::18_LD_bow_N_gram_LD_3_<e_1 ~~ e_1 ~~ ,>_[0]",  # 138
    "DependencyFeatureGenerator::18_LD_bow_N_gram_LD_3_<e_1 ~~ ( ~~ e_1>_[0]",  # 139
    "DependencyFeatureGenerator::19_LD_pos_N_gram_LD_3_<NOUN ~~ NOUN ~~ NUM>_[0]",  # 140
    "DependencyFeatureGenerator::19_LD_pos_N_gram_LD_3_<VERB ~~ ADV ~~ VERB>_[0]",  # 141
    "DependencyFeatureGenerator::23_PD_pos_N_gram_PD_2_<NOUN ~~ PROPN>_[0]",  # 142
    "DependencyFeatureGenerator::26_PD_undirected_edges_N_gram_PD_2_<appos ~~ appos>_[0]",  # 143
    "DependencyFeatureGenerator::19_LD_pos_N_gram_LD_3_<[SOURCE ~~ PUNCT ~~ NOUN>_[0]",  # 144
    "DependencyFeatureGenerator::18_LD_bow_N_gram_LD_1_<both>_[0]",  # 145
    "DependencyFeatureGenerator::18_LD_bow_N_gram_LD_1_<e_3>_[0]",  # 146
    "DependencyFeatureGenerator::18_LD_bow_N_gram_LD_2_<localize ~~ to>_[0]",  # 147
    "DependencyFeatureGenerator::18_LD_bow_N_gram_LD_2_<of ~~ e_3>_[0]",  # 148
    "DependencyFeatureGenerator::19_LD_pos_N_gram_LD_2_<PUNCT ~~ ADP>_[0]",  # 149
    "DependencyFeatureGenerator::19_LD_pos_N_gram_LD_3_<ADP ~~ PROPN ~~ NUM>_[0]",  # 150
    "DependencyFeatureGenerator::26_PD_undirected_edges_N_gram_PD_2_<pobj ~~ amod>_[0]",  # 151
    "DependencyFeatureGenerator::26_PD_undirected_edges_N_gram_PD_3_<prep ~~ pobj ~~ amod>_[0]",  # 152
    "DependencyFeatureGenerator::19_LD_pos_N_gram_LD_3_<DET ~~ NOUN ~~ PART>_[0]",  # 153
    "DependencyFeatureGenerator::26_PD_undirected_edges_N_gram_PD_2_<prep ~~ nsubj>_[0]",  # 154
    "DependencyFeatureGenerator::26_PD_undirected_edges_N_gram_PD_2_<ccomp ~~ dobj>_[0]",  # 155
    "DependencyFeatureGenerator::26_PD_undirected_edges_N_gram_PD_3_<pobj ~~ prep ~~ nsubj>_[0]",  # 156
    "DependencyFeatureGenerator::18_LD_bow_N_gram_LD_2_<with ~~ TARGET]>_[0]",  # 157
    "DependencyFeatureGenerator::26_PD_undirected_edges_N_gram_PD_1_<nmod>_[0]",  # 158
    "DependencyFeatureGenerator::26_PD_undirected_edges_N_gram_PD_1_<agent>_[0]",  # 159
    "DependencyFeatureGenerator::23_PD_pos_N_gram_PD_2_<ADV ~~ VERB>_[0]",  # 160
    "DependencyFeatureGenerator::18_LD_bow_N_gram_LD_2_<of ~~ a>_[0]",  # 161
    "DependencyFeatureGenerator::19_LD_pos_N_gram_LD_2_<PROPN ~~ VERB>_[0]",  # 162
    "DependencyFeatureGenerator::22_PD_bow_N_gram_PD_2_<involve ~~ in>_[0]",  # 163
    "DependencyFeatureGenerator::18_LD_bow_N_gram_LD_1_<protein>_[0]",  # 164
    "DependencyFeatureGenerator::19_LD_pos_N_gram_LD_3_<VERB ~~ NOUN ~~ ADP>_[0]",  # 165
    "DependencyFeatureGenerator::22_PD_bow_N_gram_PD_1_<protein>_[0]",  # 166
    "DependencyFeatureGenerator::22_PD_bow_N_gram_PD_2_<of ~~ activity>_[0]",  # 167
    "DependencyFeatureGenerator::18_LD_bow_N_gram_LD_2_<and ~~ the>_[0]",  # 168
    "DependencyFeatureGenerator::19_LD_pos_N_gram_LD_3_<NOUN ~~ PUNCT ~~ CONJ>_[0]",  # 169
    "DependencyFeatureGenerator::19_LD_pos_N_gram_LD_3_<DET ~~ NOUN ~~ NOUN>_[0]",  # 170
    "DependencyFeatureGenerator::22_PD_bow_N_gram_PD_2_<[SOURCE ~~ protein>_[0]",  # 171
    "DependencyFeatureGenerator::19_LD_pos_N_gram_LD_3_<DET ~~ VERB ~~ NOUN>_[0]",  # 172
    "DependencyFeatureGenerator::26_PD_undirected_edges_N_gram_PD_2_<conj ~~ nsubj>_[0]",  # 173
    "DependencyFeatureGenerator::18_LD_bow_N_gram_LD_2_<e_3 ~~ TARGET]>_[0]",  # 174
    "DependencyFeatureGenerator::26_PD_undirected_edges_N_gram_PD_2_<nsubj ~~ dobj>_[0]",  # 175
    "DependencyFeatureGenerator::19_LD_pos_N_gram_LD_3_<PUNCT ~~ ADP ~~ NOUN>_[0]",  # 176
    "DependencyFeatureGenerator::22_PD_bow_N_gram_PD_1_<tag>_[0]",  # 177
    "DependencyFeatureGenerator::26_PD_undirected_edges_N_gram_PD_2_<advmod ~~ dobj>_[0]",  # 178
    "DependencyFeatureGenerator::26_PD_undirected_edges_N_gram_PD_3_<advmod ~~ dobj ~~ compound>_[0]",  # 179
    "DependencyFeatureGenerator::18_LD_bow_N_gram_LD_1_<gene>_[0]",  # 180
    "DependencyFeatureGenerator::22_PD_bow_N_gram_PD_2_<[SOURCE ~~ by>_[0]",  # 181
    "DependencyFeatureGenerator::19_LD_pos_N_gram_LD_3_<NOUN ~~ NOUN ~~ PROPN>_[0]",  # 182
    "DependencyFeatureGenerator::19_LD_pos_N_gram_LD_3_<NOUN ~~ PROPN ~~ PROPN>_[0]",  # 183
    "DependencyFeatureGenerator::22_PD_bow_N_gram_PD_1_<activate>_[0]",  # 184
    "DependencyFeatureGenerator::18_LD_bow_N_gram_LD_1_<require>_[0]",  # 185
    "DependencyFeatureGenerator::18_LD_bow_N_gram_LD_1_<biogenesis>_[0]",  # 186
    "DependencyFeatureGenerator::18_LD_bow_N_gram_LD_1_<modulates>_[0]",  # 187
```

```
    "DependencyFeatureGenerator::18_LD_bow_N_gram_LD_1_<glycosylation>_[0]",  # 188
    "DependencyFeatureGenerator::19_LD_pos_N_gram_LD_3_<NOUN ~~ ADJ ~~ NOUN>_[0]",  # 189
    "DependencyFeatureGenerator::22_PD_bow_N_gram_PD_2_<expression ~~ TARGET]>_[0]",  # 190
    "DependencyFeatureGenerator::18_LD_bow_N_gram_LD_2_<and ~~ TARGET]>_[0]",  # 191
    "DependencyFeatureGenerator::19_LD_pos_N_gram_LD_3_<PUNCT ~~ CONJ ~~ TARGET]>_[0]",  # 192
    "DependencyFeatureGenerator::23_PD_pos_N_gram_PD_3_<NOUN ~~ NOUN ~~ ADP>_[0]",  # 193
    "DependencyFeatureGenerator::19_LD_pos_N_gram_LD_2_<DET ~~ ADV>_[0]",  # 194
    "DependencyFeatureGenerator::19_LD_pos_N_gram_LD_3_<VERB ~~ VERB ~~ PART>_[0]",  # 195
    "DependencyFeatureGenerator::22_PD_bow_N_gram_PD_2_<be ~~ protein>_[0]",  # 196
    "DependencyFeatureGenerator::18_LD_bow_N_gram_LD_1_<induce>_[0]",  # 197
    "DependencyFeatureGenerator::18_LD_bow_N_gram_LD_2_<e_2 ~~ and>_[0]",  # 198
    "DependencyFeatureGenerator::18_LD_bow_N_gram_LD_2_<and ~~ inhibit>_[0]",  # 199
    "DependencyFeatureGenerator::22_PD_bow_N_gram_PD_2_<retention ~~ inhibit>_[0]",  # 200
    "DependencyFeatureGenerator::23_PD_pos_N_gram_PD_3_<ADP ~~ ADP ~~ TARGET]>_[0]",  # 201
    "DependencyFeatureGenerator::26_PD_undirected_edges_N_gram_PD_3_<nsubj ~~ dobj ~~ compound>_[0]",
# 202
    "DependencyFeatureGenerator::26_PD_undirected_edges_N_gram_PD_2_<compound ~~ conj>_[0]",  # 203
    "DependencyFeatureGenerator::26_PD_undirected_edges_N_gram_PD_2_<dobj ~~ advcl>_[0]",  # 204
    "DependencyFeatureGenerator::26_PD_undirected_edges_N_gram_PD_3_<pobj ~~ prep ~~ dobj>_[0]",  #
205
    "DependencyFeatureGenerator::22_PD_bow_N_gram_PD_2_<function ~~ TARGET]>_[0]",  # 206
    "DependencyFeatureGenerator::19_LD_pos_N_gram_LD_3_<VERB ~~ ADJ ~~ TARGET]>_[0]",  # 207
    "DependencyFeatureGenerator::26_PD_undirected_edges_N_gram_PD_2_<nmod ~~ pobj>_[0]",  # 208
    "DependencyFeatureGenerator::23_PD_pos_N_gram_PD_3_<ADP ~~ PROPN ~~ TARGET]>_[0]",  # 209
    "DependencyFeatureGenerator::18_LD_bow_N_gram_LD_3_<[SOURCE ~~ be ~~ require>_[0]",  # 210
    "DependencyFeatureGenerator::19_LD_pos_N_gram_LD_3_<PUNCT ~~ ADV ~~ PUNCT>_[0]",  # 211
    "DependencyFeatureGenerator::18_LD_bow_N_gram_LD_1_<function>_[0]",  # 212
    "DependencyFeatureGenerator::18_LD_bow_N_gram_LD_2_<of ~~ e_1>_[0]",  # 213
    "DependencyFeatureGenerator::18_LD_bow_N_gram_LD_3_<- ~~ independent ~~ function>_[0]",  # 214
    "DependencyFeatureGenerator::22_PD_bow_N_gram_PD_3_<[SOURCE ~~ function ~~ require>_[0]",  # 215
    "DependencyFeatureGenerator::22_PD_bow_N_gram_PD_2_<of ~~ function>_[0]",  # 216
    "DependencyFeatureGenerator::22_PD_bow_N_gram_PD_3_<[SOURCE ~~ of ~~ function>_[0]",  # 217
    "DependencyFeatureGenerator::26_PD_undirected_edges_N_gram_PD_3_<pobj ~~ prep ~~ nsubjpass>_[0]",
# 218
    "DependencyFeatureGenerator::26_PD_undirected_edges_N_gram_PD_2_<pobj ~~ nmod>_[0]",  # 219
    "DependencyFeatureGenerator::26_PD_undirected_edges_N_gram_PD_3_<prep ~~ pobj ~~ nmod>_[0]",  #
220
    "DependencyFeatureGenerator::19_LD_pos_N_gram_LD_3_<PUNCT ~~ ADP ~~ VERB>_[0]",  # 221
    "DependencyFeatureGenerator::18_LD_bow_N_gram_LD_1_<signal>_[0]",  # 222
    "DependencyFeatureGenerator::22_PD_bow_N_gram_PD_2_<[SOURCE ~~ signal>_[0]",  # 223
    "DependencyFeatureGenerator::26_PD_undirected_edges_N_gram_PD_2_<amod ~~ nsubj>_[0]",  # 224
    "DependencyFeatureGenerator::22_PD_bow_N_gram_PD_1_<show>_[0]",  # 225
    "DependencyFeatureGenerator::26_PD_undirected_edges_N_gram_PD_2_<dobj ~~ ccomp>_[0]",  # 226
    "DependencyFeatureGenerator::18_LD_bow_N_gram_LD_1_<ubiquitination>_[0]",  # 227
    "DependencyFeatureGenerator::22_PD_bow_N_gram_PD_1_<ubiquitination>_[0]",  # 228
    "DependencyFeatureGenerator::23_PD_pos_N_gram_PD_3_<[SOURCE ~~ ADP ~~ TARGET]>_[0]",  # 229
    "DependencyFeatureGenerator::18_LD_bow_N_gram_LD_1_<activation>_[0]",  # 230
    "DependencyFeatureGenerator::19_LD_pos_N_gram_LD_3_<NOUN ~~ VERB ~~ PROPN>_[0]",  # 231
    "DependencyFeatureGenerator::19_LD_pos_N_gram_LD_3_<NUM ~~ NOUN ~~ ADP>_[0]",  # 232
    "DependencyFeatureGenerator::22_PD_bow_N_gram_PD_1_<region>_[0]",  # 233
    "DependencyFeatureGenerator::19_LD_pos_N_gram_LD_3_<ADJ ~~ ADP ~~ PROPN>_[0]",  # 234
    "DependencyFeatureGenerator::26_PD_undirected_edges_N_gram_PD_3_<nsubj ~~ advcl ~~
nsubjpass>_[0]",  # 235
    "DependencyFeatureGenerator::18_LD_bow_N_gram_LD_3_<e_1 ~~ , ~~ the>_[0]",  # 236
    "DependencyFeatureGenerator::26_PD_undirected_edges_N_gram_PD_2_<advcl ~~ prep>_[0]",  # 237
    "DependencyFeatureGenerator::18_LD_bow_N_gram_LD_2_<[SOURCE ~~ from>_[0]",  # 238
    "DependencyFeatureGenerator::22_PD_bow_N_gram_PD_2_<into ~~ TARGET]>_[0]",  # 239
    "DependencyFeatureGenerator::19_LD_pos_N_gram_LD_3_<ADP ~~ ADJ ~~ TARGET]>_[0]",  # 240
    "DependencyFeatureGenerator::19_LD_pos_N_gram_LD_2_<ADV ~~ TARGET]>_[0]",  # 241
    "DependencyFeatureGenerator::18_LD_bow_N_gram_LD_1_<this>_[0]",  # 242
    "DependencyFeatureGenerator::19_LD_pos_N_gram_LD_3_<DET ~~ ADJ ~~ ADJ>_[0]",  # 243
    "DependencyFeatureGenerator::19_LD_pos_N_gram_LD_3_<ADP ~~ PROPN ~~ PUNCT>_[0]",  # 244
    "DependencyFeatureGenerator::18_LD_bow_N_gram_LD_1_<compromise>_[0]",  # 245
    "DependencyFeatureGenerator::22_PD_bow_N_gram_PD_1_<fuse>_[0]",  # 246
    "DependencyFeatureGenerator::26_PD_undirected_edges_N_gram_PD_2_<acl ~~ acomp>_[0]",  # 247
    "DependencyFeatureGenerator::19_LD_pos_N_gram_LD_3_<NOUN ~~ PUNCT ~~ PUNCT>_[0]",  # 248
```

```
    "DependencyFeatureGenerator::18_LD_bow_N_gram_LD_1_<vesicle>_[0]",  # 249
    "DependencyFeatureGenerator::18_LD_bow_N_gram_LD_1_<dispensable>_[0]",  # 250
    "DependencyFeatureGenerator::18_LD_bow_N_gram_LD_2_<be ~~ dispensable>_[0]",  # 251
    "DependencyFeatureGenerator::22_PD_bow_N_gram_PD_2_<dispensable ~~ for>_[0]",  # 252
    "DependencyFeatureGenerator::22_PD_bow_N_gram_PD_3_<be ~~ dispensable ~~ for>_[0]",  # 253
    "DependencyFeatureGenerator::18_LD_bow_N_gram_LD_2_<e_1 ~~ to>_[0]",  # 254
    "DependencyFeatureGenerator::18_LD_bow_N_gram_LD_1_<location>_[0]",  # 255
    "DependencyFeatureGenerator::18_LD_bow_N_gram_LD_2_<[SOURCE ~~ location>_[0]",  # 256
    "DependencyFeatureGenerator::22_PD_bow_N_gram_PD_2_<[SOURCE ~~ location>_[0]",  # 257
    "DependencyFeatureGenerator::18_LD_bow_N_gram_LD_2_<, ~~ to>_[0]",  # 258
    "DependencyFeatureGenerator::18_LD_bow_N_gram_LD_2_<, ~~ while>_[0]",  # 259
    "DependencyFeatureGenerator::26_PD_undirected_edges_N_gram_PD_2_<appos ~~ compound>_[0]",  # 260
    "DependencyFeatureGenerator::19_LD_pos_N_gram_LD_3_<PUNCT ~~ VERB ~~ TARGET]>_[0]",  # 261
    "DependencyFeatureGenerator::18_LD_bow_N_gram_LD_1_<important>_[0]",  # 262
    "DependencyFeatureGenerator::22_PD_bow_N_gram_PD_1_<morphology>_[0]",  # 263
    "DependencyFeatureGenerator::26_PD_undirected_edges_N_gram_PD_3_<pobj ~~ amod ~~ npadvmod>_[0]",
# 264
    "DependencyFeatureGenerator::18_LD_bow_N_gram_LD_1_<defect>_[0]",  # 265
    "DependencyFeatureGenerator::18_LD_bow_N_gram_LD_1_<cohesin>_[0]",  # 266
    "DependencyFeatureGenerator::18_LD_bow_N_gram_LD_1_<assembly>_[0]",  # 267
    "DependencyFeatureGenerator::22_PD_bow_N_gram_PD_3_<[SOURCE ~~ determine ~~ localize>_[0]",  #
268
    "DependencyFeatureGenerator::18_LD_bow_N_gram_LD_2_<[SOURCE ~~ TARGET]>_[0]",  # 269
    "DependencyFeatureGenerator::18_LD_bow_N_gram_LD_1_<significantly>_[0]",  # 270
    "DependencyFeatureGenerator::18_LD_bow_N_gram_LD_2_<be ~~ significantly>_[0]",  # 271
    "DependencyFeatureGenerator::22_PD_bow_N_gram_PD_2_<at ~~ have>_[0]",  # 272
    "DependencyFeatureGenerator::22_PD_bow_N_gram_PD_3_<[SOURCE ~~ at ~~ have>_[0]",  # 273
    "DependencyFeatureGenerator::22_PD_bow_N_gram_PD_3_<[SOURCE ~~ recruitment ~~ of>_[0]",  # 274
    "DependencyFeatureGenerator::26_PD_undirected_edges_N_gram_PD_3_<prep ~~ nsubj ~~ prep>_[0]",  #
275
    "DependencyFeatureGenerator::18_LD_bow_N_gram_LD_3_<[SOURCE ~~ domain ~~ of>_[0]",  # 276
    "DependencyFeatureGenerator::22_PD_bow_N_gram_PD_3_<[SOURCE ~~ domain ~~ of>_[0]",  # 277
    "DependencyFeatureGenerator::22_PD_bow_N_gram_PD_1_<reconfiguring>_[0]",  # 278
    "DependencyFeatureGenerator::22_PD_bow_N_gram_PD_2_<division ~~ TARGET]>_[0]",  # 279
    "DependencyFeatureGenerator::22_PD_bow_N_gram_PD_2_<abundance ~~ TARGET]>_[0]",  # 280
    "DependencyFeatureGenerator::18_LD_bow_N_gram_LD_1_<eliminate>_[0]",  # 281
    "DependencyFeatureGenerator::22_PD_bow_N_gram_PD_2_<from ~~ through>_[0]",  # 282
    "DependencyFeatureGenerator::22_PD_bow_N_gram_PD_3_<from ~~ through ~~ TARGET]>_[0]",  # 283
    "DependencyFeatureGenerator::18_LD_bow_N_gram_LD_1_<stress>_[0]",  # 284
    "DependencyFeatureGenerator::18_LD_bow_N_gram_LD_2_<[SOURCE ~~ stress>_[0]",  # 285
    "DependencyFeatureGenerator::22_PD_bow_N_gram_PD_1_<stress>_[0]",  # 286
    "DependencyFeatureGenerator::26_PD_undirected_edges_N_gram_PD_3_<appos ~~ nsubj ~~ dobj>_[0]",  #
287
    "DependencyFeatureGenerator::22_PD_bow_N_gram_PD_3_<localization ~~ disrupt ~~ by>_[0]",  # 288
    "DependencyFeatureGenerator::18_LD_bow_N_gram_LD_3_<e_1 ~~ to ~~ TARGET]>_[0]",  # 289
    "DependencyFeatureGenerator::18_LD_bow_N_gram_LD_2_<that ~~ u>_[0]",  # 290
    "DependencyFeatureGenerator::18_LD_bow_N_gram_LD_2_<require ~~ e_1>_[0]",  # 291
    "DependencyFeatureGenerator::19_LD_pos_N_gram_LD_2_<PRON ~~ NUM>_[0]",  # 292
    "DependencyFeatureGenerator::18_LD_bow_N_gram_LD_3_<show ~~ that ~~ u>_[0]",  # 293
    "DependencyFeatureGenerator::18_LD_bow_N_gram_LD_3_<first ~~ require ~~ e_1>_[0]",  # 294
    "DependencyFeatureGenerator::19_LD_pos_N_gram_LD_3_<ADP ~~ PRON ~~ NUM>_[0]",  # 295
    "DependencyFeatureGenerator::19_LD_pos_N_gram_LD_3_<NUM ~~ ADV ~~ VERB>_[0]",  # 296
    "DependencyFeatureGenerator::22_PD_bow_N_gram_PD_2_<show ~~ require>_[0]",  # 297
    "DependencyFeatureGenerator::18_LD_bow_N_gram_LD_3_<[SOURCE ~~ to ~~ mediate>_[0]",  # 298
    "IsSpecificProteinType::40_is_marker_[0]",  # 299
    "LocalizationRelationsRatios::50_corpus_unnormalized_total_background_loc_rels_ratios_[0]",  #
300
    "LocalizationRelationsRatios::58_SwissProt_normalized_exists_relation_[0]",  # 301
]
```

Fig. S3. PR-curve analysis. *LocText* vs. *Baseline*, using manually-annotated entities. The maximum recall for both methods is 74%. The *Baseline* is shown as a single point (no decision value). A two-sample two-tailed t-test was performed to determine whether the methods' difference in F-Measure, F(*LocText*)=79%±3 vs. F(*Baseline*)=74%±3, was significant. The t-statistic was significant at the 99% confidence level, t(3998)=28.04, p=3.99e-165.

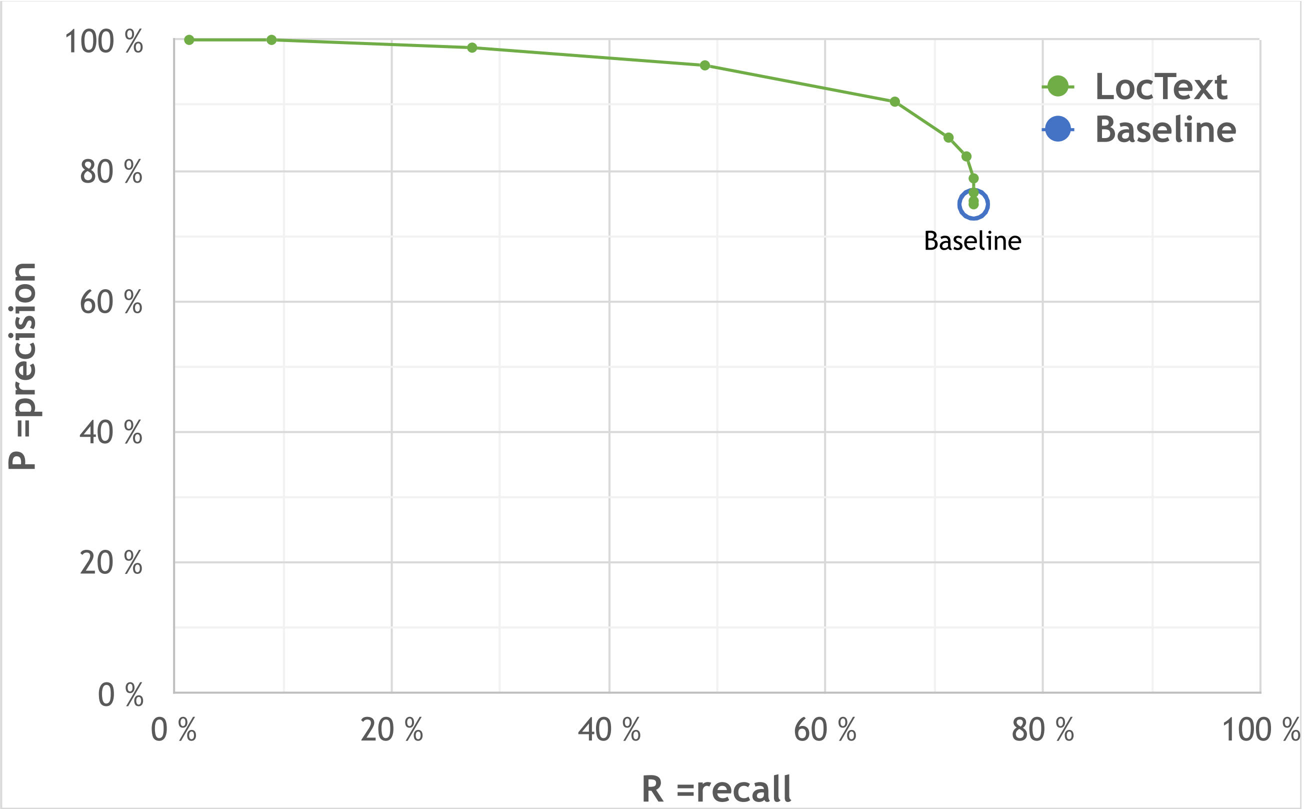

Supplement: Additional file 1 — Supporting online material. PDF document with supplemental figures and tables (Fig. S1-S3, Tables S1-S2), one per page. (PDF 238 kb) [file 12859_2018_2021_MOESM1_ESM.pdf]
